# A Comparison of Outcomes from *In Vitro* Fertilization Cycles Stimulated with Follicle Stimulating Hormone Plus either Recombinant Luteinizing Hormone or Human Menopausal Gonadotropins in Subjects Treated with Long Gonadotropin Releasing Hormone Agonist Protocols

**DOI:** 10.22074/ijfs.2017.4759

**Published:** 2017-02-16

**Authors:** Nathalie Bleau, Mohammed Agdi, WeonYoung Son, SeangLin Tan, Michael H Dahan

**Affiliations:** 1 Department of Obstetrics and Gynecology, McGill University, Montreal, Quebec, Canada; 2 Department of Reproductive Endocrinology and Infertility, McGill Reproductive Center, McGill University, Montreal, Quebec, Canada

**Keywords:** Luteinizing Hormone, *In Vitro* Fertilization, Human Menopausal Gonadotropins, Ovarian Stimulation

## Abstract

**Background:**

This study compared rates of pregnancy and *in vitro* fertilization (IVF) parameters in subjects stimulated with follicle stimulating hormone (FSH) plus either recombinant human luteinizing hormone (r-LH) or human menopausal gonadotropin (hMG) in a long gonadotropin releasing hormone (GnRH) agonist IVF protocol.

**Materials and Methods:**

This cohort study enrolled patients who underwent IVF stimu- lation with a long GnRH agonist protocol and received FSH plus r-LH or hMG. Outcomes measured included: FSH and LH doses, number of oocytes and embryos obtained, pregnancy rate per cycle, and clinical pregnancy rate per cycle. Stepwise logistic regression was performed on continuous and categorical variables to control for confounding effects between all variables analyzed.

**Results:**

There were 122 patients who underwent 122 IVF cycles with long GnRH agonist protocols. Similar baseline parameters existed between groups. Patients that received r-LH required a lower FSH dose (3207 ± 1300 IU) for stimulation compared to the group that received hMG (4213 ± 1576 IU, P=0.0001). The LH dose was also lower in these patients (1332 ± 587 IU) compared to the patients who received hMG (1938 ± 1110 IU, P=0.0001). The number of days of stimulation did not differ between groups (P=1.0). The group that received r-LH also had statistically higher numbers of oocytes (14.4 ± 6.3) and embryos (7.9 ± 4.8) compared to the hMG group with 11.0 ± 5.3 oocytes and 6.0 ± 3.7 embryos. Pregnancy rates per cycle start were higher for patients in the r-LH group (49%) compared to the hMG group (27%, P=0.025). Patients that received r-LH had higher implantation rates (62%) compared to the hMG group (33%, P=0.001). The r-LH group had a higher trend toward clinical pregnancy rates per cycle start (39%) compared to the hMG group (25%, P=0.065).

**Conclusion:**

r-LH may offer benefits compared to hMG when combined with FSH for ovarian stimulation in long GnRH agonist protocols in good responders. Prospective studies should be undertaken to confirm these results.

## Introduction

Multiple studies and meta-analyses have reported the importance of luteinizing hormone (LH) or LH mediated activity for *in vitro* fertilization (IVF) stimulation cycles (1-4). More precisely, administration of human menopausal gonadotropin (hMG) has led to increased pregnancy, clinical pregnancy, and live birth rates compared to recombinant follicule-stimulating hormone (r- FSH) alone (1, 2, 5). LH is available in two forms, recombinant (r-LH) or in hMG, which contains human chorionic gonadotropin (hCG) that acts as an LH analogue. Few studies have evaluated the role of different types of LH stimulation by comparing r-LH to HMG, which may yield subtle differences. A previous study performed at the McGill Reproductive Center compared subjects who received r-LH and r-FSH to subjects that received hMG alone in women with good or poor ovarian reserve. In subjects with good ovarian reserve, the r-LH group had higher numbers of oocyte and embryos, increased pregnancy rates per cycle, and overall higher clinical pregnancy rates which showed a potential benefit for r-LH stimulation (6). However, no distinction was made between IVF protocols.

The current study compared stimulation parameters, pregnancy and clinical pregnancy rates of patients with normal ovarian reserve parameters treated with a long gonadotropin releasing hormone (GnRH) agonist protocol and received r-LH to those treated with hMG that contained hCG as an LH analogue. Both groups of participants also received daily FSH stimulation.

## Materials and Methods

We performed a cohort study from data collected at the McGill Reproductive Center. An analysis of IVF cycles for a two-year period was undertaken to identify all patients treated at our institution that met the inclusion criteria. To be included in the study patients received FSH and either r-LH or HMG but not both forms of LH stimulation. Patients with maximum serum baseline FSH levels under 10 IU/L (drawn menstrual cycle days 2 to 5 inclusively) and baseline follicle counts of 6 follicles or more determined by transvaginal ultrasound (TVUS) as assessed on menstrual cycle days 2 to 5, inclusively, initiated treatment with a long GnRH agonist down-regulation protocol (n=122). A total of 65 women received r-LH whereas 57 received hMG. Cycles were excluded from analysis if the patient had hyperprolactinemia (morning fasting prolactin greater than 26 ng/mL), thyroid abnormalities (TSH below 0.39 or above 4.0 μIU/ mL), hypothalamic pituitary dysfunction, and ovarian failure (FSH below 2 IU/L or abover 20 IU/L and estradiol <66 pg/mL). The McGill University Committee for the Protection of Human Research Subjects approved this data collection. All subjects were de-identified in the database. Patients were allotted to their respective treatment regimens by clinic staff to maintain equivalent rates for prescription of different drugs produced by competing pharmaceutical companies. Patients that received hMG (Repronex, Ferring Canada, North York, ON) also received either follitropin beta (64%, Merck Canada, Inc., Pointe-Claire, QC), follitropin alfa (20%, EMD Serono Canada, Mississauga, ON), or purified urofollitropin (16%, Ferring Canada, North York, ON). All patients that received r-LH were treated with follitropin alfa (EMD Serono Canada, Mississauga, ON).

Patients treated with a GnRH agonist long downregulation protocol initially received stimulation with 112.5 to 225 units of FSH daily at the discretion of their treating physician. The physician selected the dose based on parameters of ovarian reserve noted during the planning of the cycle. After 5 days of FSH stimulation, we reassessed the doses which were titrated up or down depending on serum estradiol levels, as well as the numbers and diameters of follicles noted. Subsequently, patients were monitored at 1 to 3 day intervals with serum estradiol levels and transvaginal ultrasonographic follicle monitoring. Patients were prescribed LH activity such that the ratio of FSH to LH was 3:1 to 2:1 at the discretion of their treating physician. We measured peak serum estradiol levels and either 10000 IU or 5000 IU of hCG were given based on our center’s protocol 35 hours prior to egg retrieval. We followed the McGill Reproductive Center protocol for egg retrieval and embryo transfer using either a Cook (Cook Canada, Inc., Stouffville, ON) 17-gauge single lumen needle or a 16-gauge double lumen flushing needle and warmed saline flush. Pressure for aspiration was kept at 145 mmHg by a Cook Vacuum Pump (KMar 8200, Cook, Australia).

At 17 to 19 hours after insemination, embryo fertilization was evaluated for the presence of two pronuclei and two polar bodies. The zygotes were transferred to an IVF cleavage medium for further culture (Cook, Australia). The embryos were evaluated on days 2 (41-43 hours after insemination) and 3 (65-67 hours after insemination). Quality of development of the embryos was assessed according to the regularity of blastomeres, the percentage and pattern of anucleate fragments, and dysmorphic characteristics. Good quality embryos on day 2 had at least 2 cells and by day 3, they at least 6 cells with less than 20% anucleate fragments and no apparent morphological anomalies. Embryos were considered low quality if they showed blastomere multi-nucleation, poor cell adhesion, uneven cell division, and cytoplasmic anomalies. We transferred embryos of best quality based on cell number, degree of fragmentation, symmetry of blastomeres, degree of compaction, clarity and texture. Depending on age and physician orders, the transfer was performed on day 2 or 3.

Embryos were transferred under trans-abdominal ultrasound guidance and placed 2.5 to 1.5 cm from the uterine fundus using a Wallace embryo replacement catheter (Smith Medical International Ltd., UK). The number of embryos transferred varied between 2-5 depending on the patient’s age, embryo quality, and previous number of unsuccessful IVF cycles. Decisions were based according to recommendations by the American Society for Reproductive Medicine Committee Opinion (7). Progesterone was prescribed for luteal phase support per the center’s protocol. We defined pregnancy as a single serum hCG level of ≥20 IU/L measured 16 days after egg retrieval. Clinical pregnancy was defined as an intra-uterine positive fetal heartbeat seen on TVUS at 6-7 weeks of gestational age.

### Statistical analysis

Statistical analysis was done using SPSS 11.0 (SPSS Inc., Chicago, IL, USA). Continuous variables were evaluated for normal distribution using the Kolmogorov-Smirnov test. All continuous variables were normally distributed. We performed stepwise logistic regression on continuous and categorical variables to control for multiplicity and confounding effects. Rates for causes of infertility were analyzed by chi-squared tests. Data are presented as means ± SD with statistical significance as a two-sided P≤0.05. Controlled variables included patient age, basal serum FSH level, basal serum estradiol level, antral follicle count, previous pregnancies, previous full term pregnancies, previous miscarriages, previous IVF cycles, total LH and FSH stimulation doses, days of LH and FHS stimulation, as well as the number of oocytes and embryos obtained.

### Results

There were no cancelled cycles in any of the patients. Patients were good responders; hence, we did not anticipate any poor response. Ovarian hyperstimulation syndrome was avoided in all cases. No statistical differences in pregnancy rate (P=0.432) or clinical pregnancy rate (P=0.381) existed among the cycles that used follitropin alfa, follitropin beta or urofollitropin, which was combined in all cases with hMG. This result supported the combined analysis of these results. A comparison of patient demographics in subjects treated with the long GnRH agonist protocol is presented in Table 1. The two groups did not differ in any of the baseline characteristics studied, including basal serum FSH level, basal serum estradiol level, and baseline follicle count. The number of embryos transferred did not differ between r-LH (2.2 ± 0.6) versus hMG (2.3 ± 0.6, P=0.36).

**Table 1:** Patient demographics (mean ± SD)


Demographic	r-LHn=65	hMGn=57	P value

Age (Y)	35.2 ± 4.2	36.0 ± 4.4	0.412
Basal serum FSH (IU/L)	7.7 ± 1.5	7.2 ± 1.7	0.134
Basal serum estradiol (pmol/L)	167 ± 85	183 ± 77	0.292
Antral follicle count	18 ± 10	16 ± 6	0.200
Previous pregnancies	0.8 ± 1.2	1.0 ± 1.2	0.833
Previous full term pregnancies	0.1 ± 0.4	0.3 ± 0.8	0.274
Previous miscarriages	0.7 ± 1.1	0.7 ± 0.9	0.701
Previous IVF cycles at McGill	1.7 ± 0.9	1.9 ± 1.1	0.203
Previous IVF cycles elsewhere	0.4 ± 1.0	0.3 ± 0.9	0.543


Analysis performed with stepwise logistic regression. r-LH; Recombinant human luteinizing hormone, hMG; Human menopausal gonadotropin, IVF; *In vitro* fertilization, and FSH; Follicle stimulating hormone.

There were similar rates for causes of infertility in subjects treated with the long GnRH agonist protocol that received either r-LH or hMG (P=0.469).

A comparison of the r-LH or hMG groups showed that the rates of male factor infertility were 54% (r-LH) and 39% (hMG), the unexplained infertility rates were 32% (r-LH) and 33% (hMG), the rates of endometriosis were 4% (r-LH) and 12% (hMG), and the anovulation rates were 2% in both groups. Tubal factor infertility did not occur in either group.

Table 2 shows treatment outcomes for the r-LH and hMG groups when treated with the long GnRH antagonist protocol. Patients treated with r-LH had a higher pregnancy rate per cycle start (P=0.0250) and implantation rate (P=0.001) after controlling for patient age, baseline FSH and estradiol levels, antral follicle count, previous pregnancies, full term deliveries and spontaneous abortions, number of previous IVF cycles, dose of FSH and LH administered, as well as days of stimulation. Patients treated with r-LH had a trend towards a higher clinical pregnancy rate per cycle start (P=0.0649). Patients that received r-LH compared to using hMG had more oocytes collected and more embryos created, even though the r-LH group used lower doses of FSH and LH. The number of days of stimulation did not differ between the r-LH and hMG groups.

**Table 2:** IVF cycle characteristics and treatment outcomes


	r-LHn=65	hMGn=57	P value

Total FSH dose (IU)	3207 ± 1300	4213 ± 1576	0.0001
Days of FSH	8.7 ± 2.5	9.0 ± 1.7	0.248
Total LH dose (IU)	1332 ± 587	1938 ± 1110(obtained through hCG activity)	0.0001
Days of LH	7.9 ± 2.5	7.8 ± 2.6	0.997
Oocytes obtained	14.4 ± 6.3	11.0 ± 5.3	0.0100
Embryos obtained	7.9 ± 4.8	6.0 ± 3.7	0.0290
Percent of ICSI cases per group	72%	78%	0.663
Pregnancy rate per cycle start	49%	27%	0.0250
Clinical pregnancy rate per cycle start	39%	25%	0.0649
Implantation rate	62%	33%	0.001


Analysis performed with stepwise logistic regression. IVF; *In vitro* fertilization, r-LH; Recombinant human luteinizing hormone, hMG; Human menopausal gonadotropin, FSH; Follicle stimulating hormone, LH; Luteinizing hormone, and ICSI; Intracytoplasmic sperm injection.

## Discussion

In this study, there were lower FSH and LH doses required for stimulation in the long GnRH stimulation cycle with r-LH compared to hMG. r-LH treated subjects had larger numbers of oocytes and embryos obtained compared to hMG treated subjects. Pregnancy rates per cycle start and implantation rates were higher for patients in the r-LH group compared to the hMG group. There was a trend for increased clinical pregnancy rate in the r-LH group; however, this did not reach statistical significance when controlling for confounders. We observed these findings even after controlling for patient age, baseline FSH and estradiol levels, antral follicle count, previous pregnancies, full term deliveries, spontaneous abortions, and previous number of IVF cycles.

A systematic review and meta-analysis by Coomarasamy et al. (3) determined that the use of gonadotropins with LH as well as with FSH activity delivered as urinary hMG was shown to be superior to the use of r-FSH alone in long GnRH down-regulation protocols. They showed that the use of hMG was associated with a 4% increase in live birth rates compared to r-FSH alone. Other potential benefits to LH activity might also exist. Weghofer et al. (8) compared patients who underwent long protocol stimulation with either r-FSH or hMG. They found an improvement of embryonic ploidy in patients stimulated by hMG. However, the importance of the source of that LH activity should be further investigated. LH mediated activity can be administered in two forms, hMG and r-LH. LH activity in hMG is primarily achieved through hCG that acts as an LH analogue. There exist theoretical problems with hMG. For example, the risk of injection of prions through this urinary derived product, which may discourage patients and physicians from its use (9). As well, *in vitro* studies have demonstrated that r-LH and hCG result in different gene activation of the ovarian cumulous cells and endometrium (10). Therefore, r-LH may confer different beneficial effects than hMG. This difference in the endometrium may partially explain the increase in implantation seen with embryos achieved after r-LH as opposed to hMG treated cycles.

To date, few *in vivo* studies have been published. Hence, it is unclear which group of patients would benefit most from r-LH. Moro et al. (11), in a randomized controlled trial that enrolled patients over 35 years of age, found no benefit to r-LH over highly purified hMG. A study conducted in the McGill Reproductive Center observed no benefits between subjects with extremely poor ovarian reserve (baseline follicle counts less than 6) who received r-LH and r-FSH compared to subjects who received hMG. However, r-LH was found to be advantageous in terms of pregnancy and clinical pregnancy rates compared to hMG in patients with good ovarian reserve. No distinction was made between the different IVF protocols used (long agonist versus microdose flair) (6). A study by Requena et al. (12) compared endocrine profiles of 50 oocyte donors that received either r-LH plus r-FSH together or hMG and urinary FSH. Although there were more oocytes retrieved in the r-LH plus r-FSH group, a lower proportion were in metaphase II. Serum steroid levels did not differ on the day of triggering. In recipients, the implantation and ongoing pregnancy rates were the same in both groups (46.1%). However, as the recipients were not subject to LH stimulation, the difference observed between these results and the current study could be related to the possibility that LH might have a beneficial effect at the level of the endometrium as well. Conversely, a multicenter randomized controlled trial performed in Italy assessed outcomes for two groups of patients who underwent IVF using a down-regulation protocol. The first group received r-FSH plus r-LH, whereas the second group only received urinary hMG. Both groups had the same pregnancy and implantation rates. A lower cost for the IVF cycle was noted in the hMG group, as they used less FSH (13).

Our data suggests that r-LH might be beneficial compared to hCG in terms of LH mediated activity in long GnRH agonist cycles. This was a retrospective study, hence, further studies should be undertaken to confirm these results. It would have been interesting to compare follitropin-alpha and r-LH versus hMG and follitropin-alpha. The number of patients treated with this protocol was too small for comparison and should be reassessed in future studies.

## Conclusion

FSH plus r-LH may offer benefit compared to FSH plus hMG for ovarian stimulation in long GnRH-agonist protocols performed in good responders. This may occur through different stimulation of the ovarian cumulous cells or endometrium. Further studies, both larger and prospective, are needed to confirm these results.
